# Risk factors for acute compartment syndrome in one thousand one hundred and forty seven diaphyseal tibia fractures

**DOI:** 10.1007/s00264-024-06235-z

**Published:** 2024-06-18

**Authors:** Ritchie Strain, Peter Giannoudis

**Affiliations:** 1 Academic Department of Trauma & Orthopaedics, School of Medicine, Level D, University of Leeds, Clarendon WingGreat George Street, Leeds, West Yorkshire LS1 3EX UK; 2 Leeds General Infirmary, Leeds, UK

**Keywords:** Acute compartment syndrome, Trauma, Tibia fracture, Risk factors

## Abstract

**Purpose:**

Acute compartment syndrome (ACS) remains a devastating complication of orthopaedic trauma. The tibial diaphysis is especially implicated in the development of ACS, both at the time of injury and after operative management. Identification of risk factors for ACS for these distinct scenarios has been investigated in a large cohort of patients.

**Methods:**

This is a retrospective cohort study of all adults (age 18 years and older) presenting to a level 1 trauma centre with a diaphyseal tibia fracture. ACS was determined by a combination of clinical signs and symptoms and compartmental pressure monitoring. Potential risk factors were subject to univariate analysis with significant variables undergoing binary logistic regression analysis.

**Results:**

1147 tibial diaphyseal fractures over a twelve year period were studied. Age, multifragmented fracture pattern, male gender, high energy mechanism and intra- articular extension all showed a statistically significant association for ACS. Increasing body mass index (BMI) and treatment with an intramedullary nail favoured development of ACS post-operatively.

**Conclusion:**

Risk factors for the development of ACS specifically in tibial diaphyseal fractures have been highlighted. Patients managed with IMN or high BMI may warrant particular observation following operative intervention.

## Introduction

Diaphyseal tibial fractures are commonly encountered injuries for orthopaedic trauma units. They represent a spectrum of complexity from simple, closed injuries to high energy, open and multi-fragmented fractures. They can be the result of low energy fragility fractures or herald a high energy polytrauma scenario. The soft tissue envelop of the tibia can be unforgivable. This is reflected in the comparatively high proportion of open injuries and the development of acute compartment syndrome (ACS) which occurs at a rate of between 2 – 10%[1–3]. Furthermore 36% of all cases of ACS occur in the context of tibial fractures[4]. ACS is a time critical diagnosis and although diagnosis has been described using compartment pressure measurements, many orthopaedic units make this diagnosis on clinical grounds. Compartment pressure measurements are not infallable[5, 6] with no real consensus as to the diagnostic threshold and utility of compartment pressure monitoring[7]. Therefore, clinical risk factors are still an essential adjunct to diagnosis. Although a correlation between tibial fractures and ACS has been made it would be better to define this more accurately to ascertain which fracture patterns, patient demographics, injury mechanisms and management decisions play a role in the development of ACS. Previous research has identified some of these factors such as male gender, young age and open or high energy injuries[2, 8, 9]. However, studies are not specific to tibial diaphyseal fractures which represents the highest risk of ACS[2]. Many prior studies focussing on the tibia include both articular and metaphyseal segments[10–12], both of which generally have different fracture patterns, associated injuries and management principles. Therefore, it is desirable to focus particular attention on the diaphyseal segment. In addition, ACS can occur following the initial injury or may occur following surgical fixation. These patients are often grouped together as a single entity and associated risk factors analysed. However, these scenarios may represent distinct populations with differing risk factors and it would be informative to study these populations separately to elucidate any differences in the development of ACS.

The aim of this study therefore is to ascertain risk factors for ACS specific to tibial shaft fractures and whether these differ in those patients who develop ACS as a result of initial injury or surgical fixation. In addition, the impact of ACS on length of hospital stay and time to union will be investigated.

## Patients & methods

This was a retrospective cohort study. All patients aged 18 and over presenting at our institution with a diaphyseal tibial fracture either as an isolated injury or multiply injured patient were included. Sufficient follow up was taken as 12 months duration or until the patient was discharged from follow up having completed a full recovery. Demographical information along with length of stay (LOS), AO/OTA fracture classification^15^, operative details and complications were recorded. Injury mechanism was recorded in full and then classed as either low energy or high energy. High energy mechanisms included sporting injuries, road traffic collisions (RTC), fall from greater than standing height, assault or direct blows and crush injuries. Those resulting from a fall of standing height, insufficiency fractures or similar were classed as low energy. Only diaphyseal fractures were included (AO/OTA type 42), but undisplaced extension into either the proximal or distal metaphyseal segment and articular surfaces were recorded. If this extension was displaced and represented the main fracture line, then the injury was deemed an articular injury and excluded. This is in keeping with AO/OTA guidance on classification of fractures[13].

The diagnosis of ACS was made by measuring compartment pressure or clinical signs including extreme pain refectory to opioid analgesia, pain on passive stretch of compartments or muscle and soft tissue necrosis or bulging at the time of operation. When compartment pressures were used a difference of 30mmHg or less from diastolic pressure was deemed diagnostic.

Statistical analysis was performed using IBM SPSS v. 29 with factors initially undergoing univariate analysis. Dichotomous outcomes were subject to Pearson Chi square test of independence. Fischer’s exact test was used when observed counts in crosstabulations were insufficient. A Mann–Whitney U test was used to compare means due to the skew of data. Finally, those variables that showed statistical significance were subject to binary logistic regression analysis with the development of compartment syndrome as the dependent variable. Significance was assumed at p < 0.05.

This study was registered with local institutional review board with assignment number #8195.

## Results

1220 tibia fractures in 1201 patients were identified from 2008 to 2019. 73 patients were excluded due to insufficient follow up data leaving 1147 tibial fractures for further analysis. Patients comprised 32.9% females and 67.1% males with an average age of 42.62 years. A total of 368 (32.1%) injuries were open fractures. ACS was diagnosed in 58 cases (5.1%). The majority of injuries were the result of high energy mechanisms (59.3% vs 40.7%). The full breakdown of injury mechanism can be found in (Table 1).

**Table 1:** Injury mechanism and development of Compartment Syndrome

Mechanism of Injury	Number of cases (%)	Number that developed ACS (%)
Assault	29 (2.5%)	0 (0%)
Blast	2 (0.2%)	1 (50%)
Crush	45 (3.9%)	2 (4.4%)
Direct blow	31 (2.7%)	4 (12.9%)
Fall down stairs	3 (0.3%)	0 (0%)
Fall from height	74 (6.5%)	4 (5.4%)
Fall from horse	5 (0.4%)	0 (0%)
Fall from standing	450(39.2%)	8 (1.8%)
Gunshot Wound	1 (0.1%)	1 (100%)
insufficiency	1 (0.1%)	0 (0%)
Other high energy	6 (0.5%)	0 (0%)
Other low energy	9 (0.8%)	0 (0%)
Pathological	3 (0.3%)	0 (0%)
RTC	208 (18.1%)	15 (7.2%)
RTC (Pedestrian)	108 (9.4%)	8 (7.4%)
RTC (Push Bike)	13 (1.1%)	0 (0%)
Sports injury	154 (13.4%)	15 (9.7%)
Unknown	5 (0.4%)	0 (0%)
Total	1147	58 (5.1%)

Patients who developed ACS tended to be younger (26 vs 44 years), male and sustain a multi-fragmented diaphyseal tibia fracture (AO/OTA type 42C) or injuries as a result of a high energy mechanism. Open fractures did not seem to confer any statistically significant risk of ACS. In addition, fractures with an undisplaced extension into the knee joint were associated with the development of compartment syndrome (p = 0.029) (Table 2). Body Mass Index (BMI) data was available for 547 cases. Patients who developed compartment syndrome had a lower body mass index than those who did not (mean difference 1.7kg/m^2^). This trended towards, but did not achieve, statistical significance (p = 0.055).

**Table 2:** Patient demographics and fracture characteristics in patients with and without development of compartment

Compartment Syndrome					
		Yes	No	Mean difference (95% CI)	sig
Gender	Female Male	10 (2.7%) 48 (6.2%)	367 (97.3%) 722 (93.8%)		x^2^ (1) = 6.761, p = 0.009
Age		26 ± 9.7	44 ± 16	17.4 (13.08 to 21.06)	p < 0.001
BMI		25 ± 4.7	27 ± 5.4	1.7 (-0.17 to 3.46)	p = 0.055
AO/OTA (Type 42)	Simple (type A) Wedge (type B) Multifragment (type C)	30 (4.8%) 13 (3.7%) 15 (9.0%)	601 (95.2%) 337 (96.3%) 151 (91.0%)		x^2^ (2) = 6.909, p = 0.032
Open Fracture	Yes No	22 (6.0%) 36 (4.6%)	346 (94.0%) 743 (95.4%)		x^2^ (1) = 0.959, p = 0.386
High Energy	Yes No	50 (7.4%) 8 (1.7%)	630 (92.6%) 454 (98.3%)		x^2^ (1) = 18.032, p < 0.001
Articular Extension	No Ankle Knee	49 (5.1%) 5 (3.1%) 4 (15.4%)	910 (94.9%) 157 (96.9%) 22 (84.6%)		x^2^ (2) = 7.092, p = 0.029

Statistically significant variables (age, gender, fracture configuration, high energy mechanism and proximal articular extension) were all subject to binary logistic regression analysis. High energy injury mechanism and multifragmented fracture type demonstrated collinearity when subject to a correlation matrix (r = 0.176, p < 0.001) but this association was small and so both variables were included in the regression analysis.

The saturated model can be found in Table 3. Only age (being young) (adjusted OR 0.944, 95% CI 0.92 to 0.969, p < 0.001) and multifragmented fracture patterns (adjusted OR 2.31, 95% CI 1.18 to 4.53, p = 0.015) remained significant risk factors for the development of ACS.

**Table 3:** Variables associated with increased risk for developing compartment syndrome

Variables	B	S.E	Wald	df	Sig	Exp(B)	95% C.I.for EXP(B) Lower	95% C.I.for EXP(B) Upper
Age	-0.057	0.013	18.88	1	< .001	0.944	0.92	0.969
Proximal articular extension	0.961	0.613	2.459	1	0.117	2.613	0.787	8.683
High energy	0.689	0.42	2.686	1	0.101	1.992	0.874	4.541
Male	0.159	0.38	0.175	1	0.676	1.173	0.556	2.471
Multifragmented fracture	0.839	0.343	5.97	1	0.015	2.313	1.18	4.532
Constant	-1.705	0.666	6.554	1	0.01	0.182		

The median length of stay was significantly increased in those patients who developed ACS than those who did not (16 days vs 8 days, *z* = 5.503, p < 0.001).

The median time to radiological union was prolonged in patients who developed ACS compared to those who did not (189 days vs 167 days, z = 2.920, p = 0.004).

ACS developed prior to definitive management (pre-operative ACS) in 51 patients and following definitive management (post-operative ACS) in eight patients (86.4% vs 13.6%), Table 4. Fixation methods included intramedullary nail (IMN, n = 22), circular frame (n = 35), plate osteosynthesis (n = 1) and modular rail system (MRS, n = 1). Both patients treated with MRS and plate osteosynthesis were excluded from analysis to increase statistical power. Both increasing BMI and management with a reamed IMN were associated with the development of post-operative ACS.

**Table 4:** Risk factors of development of ACS prior to definitive management (pre-operative ACS) and following definitive management (post-operative ACS)

Compartment Syndrome					
		Pre-op	Post-op	Mean difference (95% CI)	sig
Gender	Female Male	9 (90%) 42 (85.7%)	1 (10%) 7 (14.3%)		x^2^ (1) = 0.130, p = 0.718
Age		30.4 ± 10.7	27.4 ± 10.8	3.0 (-5.2 to 11.2)	p = 0.465
BMI		26.4 ± 4.6	20.7 ± 1.1	5.66 (3.29 to 8.05)	p < 0.001
AO/OTA Classificatio n	Simple (type A) Wedge (type B) Multifragment (type C)	24 (77.4%) (92.9%) (100%)	7 (22.6%) 1 (7.1%) 0 (0%)		x^2^ (2) = 4.840, p = 0.089
Open Fracture	Yes No	34 (89.5%) 17 (81.0%)	4 (10.5%) 4 (19.0%)		x^2^ (1) = 0.838, p = 0.360
High Energy	Yes No	44 (86.3%) 7 (87.5%)	7 (13.7%) 1 (12.5%)		x^2^ (1) = 0.009, p = 0.925
Articular Extension	No Ankle Knee	43 (86.0%) 4 (80.0%) 4 (100.0%)	7 (14.0%) 1 (20%) 0 (0.0%)		x^2^ (2) = 0.813, p = 0.666
Smoking status (n = 50)	Yes No	18 (78.3%) 25 (92.6%)	5 (21.7%) 2 (7.4%)		Fisher's Exact p = 0.225
Definitive Managemnt (n = 57)	IMN Frame	15 (68.2%) 35 (100%)	7 (31.8%) 0 (0%)		Fisher's Exact p < 0.001

## Discussion

This study aimed to assess the risk factors for ACS exclusively following diaphyseal tibial fractures from a large sample size and whether these differed in patients developing ACS following definitive management or not.

McQueen et al. conducted a retrospective review of 1407 diaphyseal tibial fractures over a 13 year period[8]. ACS developed in 11.5% of patients. Young age proved the most significant risk factor for the development of ACS. Other risk factors including male gender, sporting injuries, occupation and treatment with an intramedullary device were all predictive of ACS.

More recently, in 2024, Ming et al. [14] reviewed the risk factors for compartment syndrome with particular focus on laboratory based results. They found that male gender, crush injuries and elevated white cell count and LDH on admission bloods were predictive of ACS. However, there was no appreciation of the operative fracture management, associated injuries or injury classification and how these relate to the risk of ACS. In addition, no distinction was made between those patients who developed ACS pre- or post-operatively, something this study aimed to appreciate.

Wuarin et al.[11] performed a retrospective review of 273 tibial fractures and attempted to accurately describe fracture patterns and locations using several radiographic measurements. Fractures that occurred more than 15cm proximal to the talar dome showed a significant association with ACS. In addition, age, closed fractures and concurrent pilon or plateau fractures also demonstrated statistical significance.

There has been two recent meta-analysis of ACS risk factors. In 2021 Mortensen conducted a meta-analysis of risk factors for ACS based on current literature[15]. The authors corroborated young age and male gender as a risk factor. Furthermore, gunshot wounds, vascular injuries and high energy injury mechanisms were regarded as risk factors. This meta-analysis was not specific to the development of ACS in tibial shaft fractures and unfortunately due to the paucity of specific studies and those with a control group, no subgroup analysis of tibial shaft fractures could be performed. In 2023, Wang et al.[16] focussed exclusively on the risk factors for ACS following tibial fractures. They reported several significant associations with ACS including age, gender, fracture characteristics and certain radiographic measurements. However, these significant risk factors are generic to all tibial fractures as the study did not discern between diaphyseal, plateau or pilon fractures. Therefore, risk factors such as tibial widening in plateau fractures will not be applicable to diaphyseal fractures. Our analysis aims to focus specifically on ACS in the diaphyseal tibia which these two large meta-analysis have not been able to.

ACS still represents a considerable burden following diaphyseal tibia fractures with a rate of 5.1% in our cohort, although this was less than previously discussed studies. In line with all other studies in the determinants of ACS, youth would appear to be the overwhelming risk factor in predicting ACS. In our study, this was followed by multifragmented fracture pattern. Gender, high energy mechanisms and intra-articular extension also demonstrated significant association with ACS in univariate analysis but failed to maintain this once age was considered. At odds with previous studies, we did not find a significant association with closed fractures or smoking status. Interestingly, patients with ACS had a lower mean body mass index than those who did not. This association trended towards statistical significance but did not achieve it (p = 0.055). This is perhaps an area that warrants further exploration. One theory may simply be that with reduced body mass index comes reduced compartment volume and therefore the threshold for ACS may be reached sooner.

The regression model was only able to account for a small proportion of positive cases in the cohort and a large proportion of cases of ACS remain unaccounted for. It is perhaps unrealistic to assume that ACS can be predicted on these parameters alone.

When assessing the timing of the development of ACS both increasing BMI and management with an IMN appeared to favour the development of ACS following definitive management. To our knowledge this is the first study to look specifically at risk factors for pre- and post-operative ACS.

The question of adverse fracture healing has been raised in the presence of ACS as early as 1987. Court-Brown et al. reported tibial fractures complicated by ACS took an average of 20 weeks longer to unite than controls[17]. This result was based on a subset of 42 patients all of whom were male and was not specific to diaphyseal tibia fractures. In 2011, Reverte et al.[18] conducted a literature review analysing 16 articles relating to fracture healing in tibial fractures complicated by ACS. By pooling patients from these studies, the authors concluded that ACS delayed healing by an average of five weeks. However, this included patients of all age groups and did not discern between fracture morphology or classification, in particular diaphyseal tibial fractures again were not considered in isolation. Our results seem to corroborate this finding. Patients who developed ACS took an average of 22 additional days to unite compared to those who did not develop ACS. Although significant, this difference was not as striking as previous studies, but does add accuracy in that it pertains to diaphyseal fractures specifically.

This study must be taken in the context of its limitations. As it is retrospective in nature, it is prone to recall and information bias. For example, smoking status was only available for 547 patients (47.7%). In addition, creating a comparative group is also challenging in retrospective studies. Given the rarity and unpredictability in which ACS occurs, this would not be feasible even in a prospective study. The study period lasted 12 years in duration and accumulated 58 cases of ACS. Therefore, a prospective study would likely have yielded much smaller numbers than this making inferential statistical analysis a challenge. Patients were mostly managed with intramedullary devices or circular frames. Only one patient was managed with plate osteosynthesis and one patient was managed with a modular rail system. This may not be representative with other institutions meaning inferences from this study may not be transferrable.

This study does however add additional evidence on the rate, risk factors and outcomes in terms of fracture union and length of stay in a large cohort of patients. Furthermore, it is specific to the diaphyseal segment of the tibia which has historically seen the highest rates of ACS compared to the metaphyseal segments.

## Data Availability

Data supporting this study cannot be made available as participants did not agree to their data being shared publicly.
